# Increased NOTCH1 expression is associated with low survival in moderate/ poor differentiated human oral squamous cell carcinoma patients

**DOI:** 10.7150/jca.87128

**Published:** 2023-09-18

**Authors:** Zuhair M. Mohammedsaleh, Mamdoh S. Moawadh, Fayez M. Saleh, Mohammed M. Jalal, Abdulaziz S Al-Otaibi, Nizar H. Saeedi, Rathinasamy Baskaran, Chih-Yang Huang, V. Bharath Kumar

**Affiliations:** 1 Department of Medical Laboratory Technology, Faculty of Applied Medical Sciences, University of Tabuk, Tabuk 71491, Saudi Arabia.; 2 Department of Medical Microbiology, Faculty of Medicine, University of Tabuk, Tabuk 71491, Saudi Arabia.; 3 Department of Bioinformatics and Medical Engineering, Asia University, Taichung, Taiwan.; 4 Department of Medical Research, China Medical University Hospital, China Medical University, Taichung 404, Taiwan.; 5 Department of Medical Laboratory Science and Biotechnology, Asia University, Taichung 413, Taiwan.

**Keywords:** Notch1, Oral cancer, tumor differentiation, IHC, mortality, survival

## Abstract

Notch deregulation has been reported in various types of cancers, including Oral squamous cell carcinomas (OSCCs). The role of Notch1 signaling in oral squamous cell carcinoma (OSCC) remains poorly understood. In this study, NOTCH1 was aberrantly expressed in human oral cancer tissues compared with that in normal marginal tissues and was associated with poor prognosis. The positive Notch 1 expression was significantly associated with poor tumor differentiation status. Kaplan-Meier survival curves revealed that elevated cytoplasmic NOTCH1 expression levels in OSCC patients were associated with poor overall survival. Moreover, multivariate COX proportional hazard models revealed that T N status, AJCC stage histological grade were independent prognostic factors for survival. Our result clearly demonstrates the oncogenic role of Notch1 in oral cancer and Notch1 may be a useful biomarker to target oral cancer patients.

## Introduction

Oral cancer is one of the most common cancers in the worldwide, according to the results shown by GLOBCAN 2012 (http://globocan.iarc.fr/)300,000 new oral cancer cases and 145,000 oral cancer-related deaths were registered in 2012 1, 2. Despite of the improved diagnostic techniques and treatments, survival rate of oral cancer patients has not been improved 3. This could be improved by identifying the molecular biomarkers 4; however, the developmental path towards a clinically suitable biomarker remains tremendously tough. Despite of the improved diagnostic techniques and treatments, survival rate of oral cancer patients has not been improved 3. This could be improved by identifying the molecular biomarkers 4, however, the developmental path towards a clinically suitable biomarker remains tremendously tough. Thus, it is essential to identify the novel molecular markers and develop effective approaches to treat OSCC patients.

Notch signaling plays a significant role in cell proliferation, differentiation, and apoptosis 5, 6. Notch families have four receptors (Notch1-4) and five ligands (Delta-like-1, Delta-like-3, Delta-like-4, Jagged1, and Jagged2) 7. NOTCH1 is one of the most commonly mutated tumor suppressor genes in Head and neck squamous cell carcinomas (HNSCCs)^8^. Notch-1 expression was found to be elevated in salivary adenoid cystic carcinoma (SACC)tissues 9, and promotes proliferation of SACC and HNSCC 9, 10. Several studies have reported tumor-suppressive role or oncogenic role of Notch signaling pathway in many cancer types 11-14 including HNSCCs 15-17. All these results suggest that the NOTCH1 pathway may have dual function in this tumor type. Thus, understanding NOTCH1 expression pattern and its function are likely to have therapeutic potential in OSCC 18, 19.

In the present study, immunohistochemical (IHC) analysis was used to examine the protein expression of NOTCH1 in oral cancer patients. We also examined the relationship between NOTCH1 protein expression and OSCC clinicopathological variables and prognosis, and whether NOTCH1 could be a prognostic biomarker in these patients.

## Materials and methods

### Patient samples

To evaluate the association of NOTCH1 expression with clinical/pathological factors and patient survival, a total of 268 oral cancer tissues were obtained from 253 male and 15 female patients (age ranged from 30 to 90 years old) were obtained from Human Bio Bank, China Medical University and Hospital, Taiwan. The main treatment was tumor removal and radical neck dissection, including post operation irradiation as well as selective patients treated with 5-fluorouracil (5-FU) and cisplatin chemotherapy. The tumors were classified according to the International Union against Cancer TNM classification system 20. All samples were snap-frozen in liquid nitrogen and stored at -80 °C until use.

### Tissue microarray and Immunohistochemistry

Tissue microarrays (TMAs) were created using the OSCC samples based on the methods outlined in previous reports 21. Two senior pathologists validated the morphology of the malignancy based on representative lesions revealed by hematoxylin and eosin (H&E) staining.

Immunohistochemistry analysis was performed according to the standard protocol as previously described 22. Tissue sections were deparaffinized and rehydrated using routine techniques. Endogenous peroxidase activity was blocked with 3% H2O2 in methanol, hydrated with gradient alcohol and phosphate-buffered saline solution, and incubated in 10 mmol/L citrate buffer (pH 6.0). Tumor sections were incubated with NOTCH1 mouse monoclonal antibody (Catalog number: CF500248; 1:50 dilution; origene, Rockville, MD, USA) in room temperature for 20 mins. After washing three times with PBS, the sections were incubated with appropriate peroxidase-labelled secondary antibodies for 30 min at room temperature. The sections were washed three times with PBS and then labelled by diaminobenzidine and counterstained with Mayer's haematoxylin, dehydrated and mounted. Two experienced pathologists independently assessed the results of immunohistochemical staining, and a final agreement was obtained for each score at a discussion microscope.

### Statistical analysis

All data were analyzed by the SAS 9.4 Software (SAS Institute, Inc.; Cary, NC, USA). Chi-square test was used to detect the importance of the clinicopathological variables of NOTCH1 protein expression and OSCC. The overall survival (OS) time, was estimated with the Kaplan-Meier method and compared using the Log rank test. Univariate and multivariate analysis was performed to confirm prognostic factors of OSCC using the Cox proportional hazard regression model 23. Statistically significant results were defined by a p value of < 0.05.

## Results

### NOTCH1 protein expression in OSCC and clinicopathological variables

**Figure 1:**
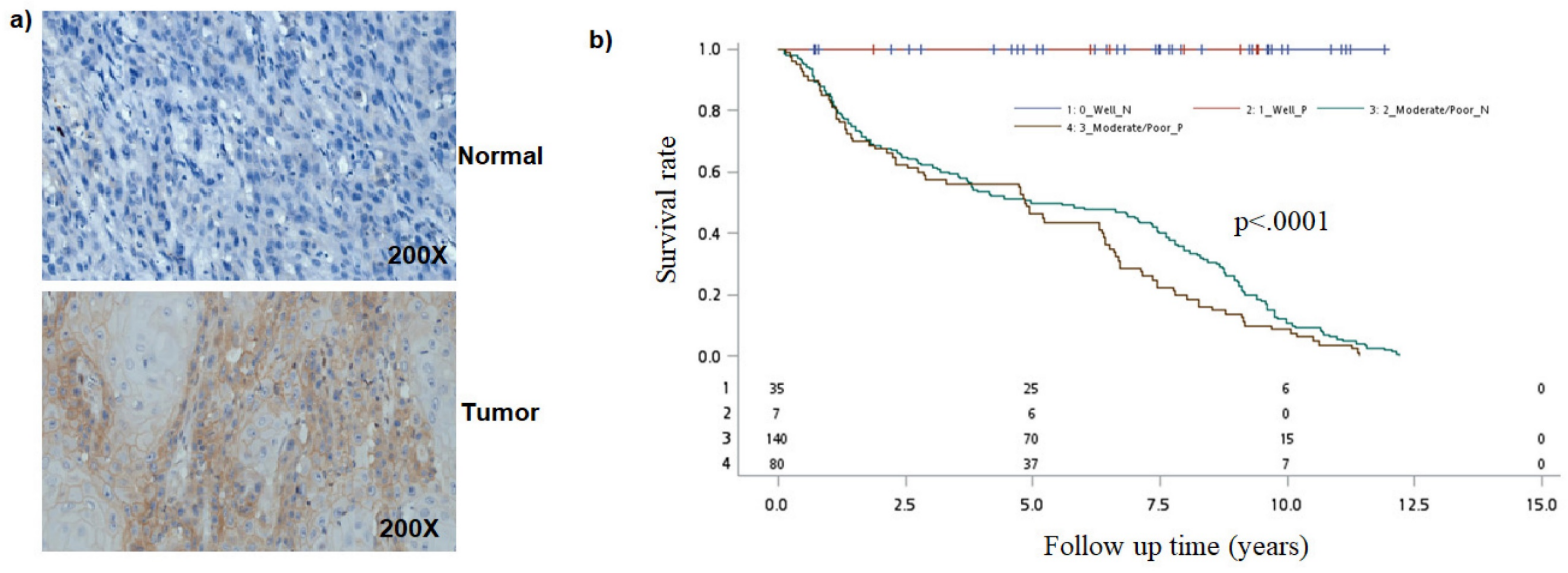
Analysis of NOTCH1 in oral cancer cells. a) Immunohistological detection of NOTCH1 in OSCC samples. b) Kaplan-Meier survival curves associated with NOTCH1 expression among oral cancer patients in Tumor differentiation. P-values obtained from log-rank tests for the homogeneity of Kaplan-Meier curves between high and low NOTCH1 expressions was 0.0009.

Earlier studies have found that Notch1 function as oncogene or tumor-suppressor 24-27. To better understand the role of the Notch pathway in oral cancer, we first analyzed for NOTCH-1 expression by immunohistochemical staining. Immunohistochemical examination revealed that Notch1 was accumulated in the cytoplasm or nuclei of human oral cancer samples (Figure 1a).

To explore the potential application of determination of Notch1 levels for early diagnosis and prognosis in oral cancer, we analyzed the correlation between Notch1 expression and patient clinicopathological features and survival. Table 1 shows the demographic and clinicopathologic characteristics of OSCC patients. Specimens from 268 patients were included in this study (253 male and 15 female patients).

Correlations between Notch1 expression and the clinicopathological characteristics of patients with oral cancer were investigated. As shown in Table 2, a significant positive correlation was observed between Notch1 expression and tumor differentiation (P<0.05), but not between Notch1 expression and TNM stage, AJCC stage and Death status (P>0.05). These results are consistent with the previous report by 28. The percentage of NOTCH1 positive cases in the poorly differentiated group was lower than that in the well/moderately differentiated group.

### Notch1 expression and survival

The effects of clinicopathologic factors and Notch1 expression on mortalityin the OSCC patient are shown in Table 3. The mortality density for patients with T III/IV, N2/N3, AJCC tumor stage (III/IV), Moderate/Poor tumor differentiation and Chemotherapy/Radiotherapy was 13.0, 24.2, 12.8, 9.5 and 13.0, respectively per 100 people-years. Higher mortality risk was observed to be related to Tumor size (III/IV), N stage (N2/N3), AJCC tumor stage (III/IV), moderate/poor tumor differentiation, and chemotherapy/radiotherapy was significantly associated with a higher mortality risk (aHR = 2.02, 3.06, 2.65, 2.73 and 3.42 respectively). The independent mortality risk for patients with negative Notch1 expression was non-significant as compared those with positive expression (95% CI, 0.76-1.64; P> 0.05).

Because NOTCH1 expression was associated with tumor differentiation, we then evaluated the combined effect of NOTCH1 expression and tumor differentiation on mortality. As compared to patients with well differentiation and negative NOTCH1 expression, the mortality hazard risk was multiplicatively enhanced among patients with Moderate/Poor tumor differentiation and positive NOTCH1 expression (aHR = 2.54, 95% CI, 1.44-11.20; *P* = 0.0081 for multiplicative interaction, Table 3). Moreover, patients with a positive Notch1 expression had a shorter overall survival (OS) time than those with a negative Notch1 expression by Kaplan-Meier analysis (*P=0.0009*; Figure 1b).

## Discussion

The role and clinical relevance of Notch1 in cancers have not been well illustrated. However, a few studies have suggested Notch1 as a tumor promoter in head and neck squamous cell carcinomas (HNSCCs) 27, 29. Though, some studies have shown that Notch function as tumor suppressor 24. This study aimed to explores the expression of NOTCH1 in oral cancer tissues and its influence on prognosis.

Notch1 is crucial in tumor progression and plays a dual role either as oncogene and tumor suppressor 30. In this present study Notch1 expression was seen in the cytoplasm of oral cancer cells, while a weak expression was found in normal tissues 4, 31-33. The relationship between Notch1 expression levels and certain clinicopathological parameters was evaluated. Our findings showed that high Notch1 protein level was associated with tumor differentiation, but not with TNM classification, tumor stage and death. Conversely, Tian J et al (2018) have found that the expression of Notch1 was positively correlated with distant metastasis (P=0.003) and tumor differentiation (P=0.031) 34.

The significant prognostic variables provide useful information for clinical treatment and it is beneficial for identifying patients who have a higher risk of disease recurrence or poor outcome. The significant prognostic variables for survival were the late-stage TN (III/IV) and Poor tumor differentiation, whereas NOTCH1 expression was not correlated with OSCC mortality. Therefore, we studied the combinatory effect of tumor differentiation and NOTCH1 expression on OSCC morality. Significant multiplicative-scale interaction between Tumor differentiation and the presence of NOTCH1 on mortality risk was identified (P=0.0081). Moreover, we found poor tumor differentiated patients with high NOTCH1 had a low survival rate at a 10 year follow up. Further research, i) using both *in vitro* and *in vivo* testing is required to support our data and assess the efficacy of NOTCH1 as a therapeutic target; ii) number of patients sample used in this study is relatively small and larger sampled are needed to validate these results.

In conclusion, high NOTCH1 expression is observed in oral cancer and more likely to worsen survival in poorly differentiated oral cancer patients. These findings emphasize oncogenic role of NOTCH1 in poorly differentiated oral cancer patients.

## Figures and Tables

**Table 1:** Clinical characteristics of oral cancer patients

Factors	No	%
Gender		
Female	15	5.6
Male	253	94.4
Age, year		
≤49	83	31
50-59	95	35.4
60-69	59	22
≥70	31	11.6
T (tumor size)		
I	68	25.4
II	84	31.3
III	21	7.8
IV	95	35.5
N (lymph node)		
N0	173	64.6
N1	33	12.3
N2	59	22
N3	3	1.1
M (metastasis)		
No	268	100
Yes	0	0
AJCC cancer stage		
I	53	19.8
II	56	20.9
III	31	11.5
IV	128	47.8
Histological grade		
Well	42	15.7
Moderate	218	81.3
Poor	8	3
**Clinical therapy**		
Radiotherapy		
No	97	38.8
Yes	161	61.2
Chemotherapy		
No	192	74.8
Yes	65	25.2

**Table 2:** Association of Notch1 expression with clinical parameters of 268 OSCC patients

	Cytoplasm			
	NOTCH1			
Factors	Negative	Positive		
	No.	No.		
	(n = 180)	(n=88)	^*^aOR(95% CI)	* P-value*
Tumor size (SD)	2.91(1.60)	2.96(1.56)		0.8
T classification				
I/II	97	55	1	
III/IV	83	33	0.70(0.42-1.18)	0.1823
N (lymph node)				
N0/N1	137	69	1	
N2/N3	43	19	0.88(0.48-1.62)	0.6851
M (metastasis)				
No	180	88	1	
Yes	0	0	ND	
AJCC cancer stage				
Early stage (I/II)	69	40	1	
Advance stage (III/IV)	111	48	0.75(0.44-1.25)	0.263
Tumor differentiation				
Well	1	7	1	
Moderate/Poor	145	81	2.77(1.17-6.51)	0.02
Death				
No	100	51	1	
Yes	80	37	0.90(0.53-1.50)	0.674

*aOR- Adjusted odds ratio was controlled for gender and age

**Table 3:** Clinicopathologic characteristics and NOTCH1 expression on mortality density and adjusted hazard ratio (aHR) among OSCC patients

Factors	No. of patient	Follow-up	No. of deaths	Mortality density^a^	aHR^b^	(95% CI)	Interaction
		(person-year)					P-value
**Overall mortality from primary malignancy to death**							
T classification							
I/II	**152**	891.25	52	5.8	1		
III/IV	**116**	501.84	65	13.0	2.02	(1.40-2.92)	0.0004
N classification							
N0/N1	**206**	1210.92	73	6.0	1		
N2/N3	**62**	182.18	44	24.2	3.06	(2.09-4.48)	<.0001
AJCC tumor stage							
I/II	**109**	715.45	30	4.2	1		
III/IV	**159**	677.64	87	12.8	2.65	(1.75-4.02)	<.0001
Tumor differentiation							
Well	**42**	259.21	9	3.5	1		
Moderate/Poor	**226**	1133.88	108	9.5	2.73	(1.38-5.39)	0.0016
Clinical therapy							
Surgery	**106**	692.67	26	3.8	1		
Chemotherapy/Radiotherapy	**162**	700.42	91	13.0	3.42	(1.95-4.69)	<.0001
NOTCH1 Expression							
Negative	**100**	1064.32	80	7.5	1		
Positive	**51**	445.2	37	8.3	1.12	(0.76-1.64)	0.7933
**Combined effect**							
Tumor differentiation/NOTCH1^c^							
Well/Negative	**35**	242.2	9	3.7	1		
Well/Positive	**7**	50.37	0	0.0	ND	ND	
Moderate/Poor/Negative	**145**	822.1	71	8.6	2.32	(1.43-10.85)	
Moderate/Poor/Positive	**81**	394.8	37	9.4	2.54	(1.44-11.20)	0.0081

^a^Mortaility density was displayed as per 100 people-years. ^b^aHR was adjusted for gender and age. ^c^Significant multiplicative-scale interaction between Tumor differentiation and the presence of NOTCH1 on mortality risk was identified (*P* for interaction, 0.0081).
